# Prolonged Activation of Virus-Specific CD8^+^T Cells after Acute B19 Infection

**DOI:** 10.1371/journal.pmed.0020343

**Published:** 2005-11-01

**Authors:** Adiba Isa, Victoria Kasprowicz, Oscar Norbeck, Andrew Loughry, Katie Jeffery, Kristina Broliden, Paul Klenerman, Thomas Tolfvenstam, Paul Bowness

**Affiliations:** **1**Institution for Medicine, Infectious Disease Unit, Center for Molecular Medicine, Karolinska Institutet, Karolinska University Hospital, Stockholm, Sweden,; **2**MRC Human Immunology Unit, Weatherall Institute of Molecular Medicine, Oxford, United Kingdom,; **3**Peter Medawar Building for Pathogen Research, Nuffield Department of Medicine, Oxford University, Oxford, United Kingdom,; **4**Department of Virology, John Radcliffe Hospital, Oxford, United Kingdom,; **5**Division of Clinical Virology, Karolinska Institutet, Karolinska University Hospital, Stockholm, Sweden,; **6**Nuffield Orthopaedic Centre NHS Trust, Oxford, United Kingdom; University of BirminghamUnited Kingdom

## Abstract

**Background:**

Human parvovirus B19 (B19) is a ubiquitous and clinically significant pathogen, causing erythema infectiosum, arthropathy, transient aplastic crisis, and intrauterine fetal death. The phenotype of CD8^+^ T cells in acute B19 infection has not been studied previously.

**Methods and Findings:**

The number and phenotype of B19-specific CD8^+^ T cell responses during and after acute adult infection was studied using HLA–peptide multimeric complexes. Surprisingly, these responses increased in magnitude over the first year post-infection despite resolution of clinical symptoms and control of viraemia, with T cell populations specific for individual epitopes comprising up to 4% of CD8^+^ T cells. B19-specific T cells developed and maintained an activated CD38^+^ phenotype, with strong expression of perforin and CD57 and downregulation of CD28 and CD27. These cells possessed strong effector function and intact proliferative capacity. Individuals tested many years after infection exhibited lower frequencies of B19-specific cytotoxic T lymphocytes, typically 0.05%–0.5% of CD8^+^ T cells, which were perforin, CD38, and CCR7 low.

**Conclusion:**

This is the first example to our knowledge of an “acute” human viral infection inducing a persistent activated CD8^+^ T cell response. The likely explanation—analogous to that for cytomegalovirus infection—is that this persistent response is due to low-level antigen exposure. CD8^+^ T cells may contribute to the long-term control of this significant pathogen and should be considered during vaccine development.

## Abstract 2

Although parvovirus B19 apparently causes an acute viral infection, it appears to induce a persistent activated CD8 ^+^ T cell response.

## Introduction

Human parvovirus B19 (B19) is a ubiquitous, single-stranded DNA virus. The 5.6-kb genome codes for only three major proteins, the two overlapping capsid proteins VP1 and VP2 and the non-structural protein NS1. B19 targets immature erythroid cells in the bone marrow after respiratory transmission. Common manifestations of the infection are the benign febrile illness erythema infectiosum followed by an acute arthropathy (in approximately 5% of children and up to 50% of adult infections) that spontaneously resolves within 3 wk. Some adult patients who develop chronic arthritis can fulfil the diagnostic criteria of rheumatoid arthritis [1,2]. Other severe manifestations include transient aplastic crisis in individuals with increased red cell turnover, and chronic anaemia in immunocompromised patients. Furthermore, infection during pregnancy is a major cause of fetal death. Clinical resolution of acute infection is associated with the emergence of antiviral IgG, which is maintained lifelong [3]. However, although antibodies are of importance, evidence for an important role for cellular immune responses in the control of B19 infection is also emerging. In some individuals with apparently intact antibody responses, virus replication continues long term [4]. Readily detectable CD8^+^ T cell responses in three asymptomatic seropositive individuals have also been observed [5]. CD8^+^ T cells play an essential role in the control of viral infections by direct killing of virus-infected cells and through cytokine secretion. After a primary viral infection, naïve antigen-specific T cells expand clonally and undergo several differentiation stages, followed by a contraction phase mediated by apoptosis. Expression of the chemokine receptor CCR7 on memory T cells has been used to divide this population into central and effector memory subsets. CCR7^+^ “central” memory lymphocytes mostly home to secondary lymphoid organs and have a high proliferative capacity in response to antigen re-encounter. CCR7^−^ “effector” memory cells home to non-lymphoid organs and are capable of rapid effector function [6]. Peptide–major histocompatibility complex multimers can be used to further quantify and phenotype T cells bearing T cell receptors of appropriate peptide/human leukocyte antigen (HLA) specificity [7,8]. Amongst “effector” memory populations, a spectrum of phenotypes exists, as assessed by surface expression of markers such as CD27, CD28, and CD57, and intracellular expression of perforin. Amongst the viral infections studied, cytomegalovirus (CMV) is associated with the emergence of the largest long-term memory populations with the most “mature” phenotype, CD27/CD28 low, CD57 high, and often perforin positive [9]. The factors that ultimately determine the phenotype and function of these populations of lymphocytes are not fully understood, but continued exposure to antigen is thought to be important. Persistent infections such as hepatitis C virus (HCV), Epstein–Barr virus (EBV), and HIV also show antiviral populations that are “effector” (CD62L or CCR7 low), but show lower levels of maturation than CMV-specific responses [7].

Its small stable genome and clear seroconversion illness make B19 an ideal viral infection in which to study the evolution of antigen-specific T cells longitudinally. Most other significant viruses in humans are either highly variable (HCV and HIV) or have extremely large genomes (EBV and CMV). In our previous analyses, we observed a surprisingly robust response to B19 in three individuals with an asymptomatic seropositive state [5]. We aimed in this study to define the origins of such responses and track a range of antiviral populations during and after acute disease.

## Methods

### Study Participants

Eleven previously healthy immunocompetent adults presenting to their general practitioner with symptoms of fever, arthralgia, fatigue, and rash were prospectively identified (B19 IgM-positive) at the Departments of Clinical Virology at the Oxford Radcliffe Hospitals, Oxford, United Kingdom, and Karolinska University Hospital, Stockholm, Sweden. The two cohorts were ascertained and studied independently. Clinical details of the patients are shown in Table 1. The timing of blood samples is given from onset of symptoms for both cohorts. B19 DNA in serum was detected by nested PCR amplifying 284 bp in the NS1 gene with a sensitivity of 10^3^ DNA copies/ml (Table 1) [5]. In addition, five healthy B19 IgG-positive, B19 IgM/DNA-negative healthy laboratory volunteers were studied as a “remotely infected” cohort. One gave a history of acute rash and arthritis 10 y previously; the remainder had no history suggestive of B19 infection and were likely infected in childhood. No interferon-γ (IFNγ)– or B19-specific T cells were detected in B19 IgG/IgM/DNA-negative healthy controls (data not shown). Peripheral blood mononuclear cells (PBMCs) were separated from heparinized blood samples within 8 h of sampling, by gradient centrifugation on Ficoll-Paque (Amersham Biosciences, Uppsala, Sweden) or Lymphoprep (Fresenius Kabi Norge, Halden, Norway). DNA was extracted from PBMCs using a QIAamp DNA Mini kit (VWR International, Stockholm, Sweden) or PURGENE DNA purification kit (Gentra Systems, Minneapolis, Minnesota, United States). B19 IgM and IgG were tested using a commercial EIA (Biotrin International, Dublin, Ireland).

**Table 1:**
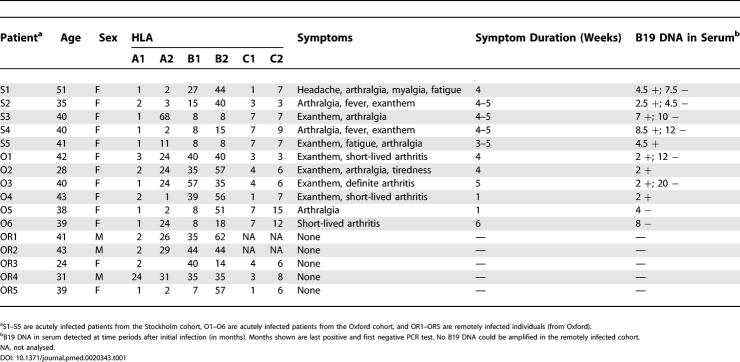
Data on Individuals Acutely and Remotely Infected with B19

Ethical approval for the study was obtained from the local ethical committee at Huddinge University Hospital, Karolinska Institutet, Stockholm, Sweden, and from the Oxfordshire Clinical Research Ethics Committee (CO2.113), Oxford, United Kingdom, and informed patient consent was obtained.

### HLA Tissue Typing

HLA class I genotyping was performed using multiplex PCR on DNA extracted from PBMCs (ABC SSP Unitray, Dynal Biotech, Oslo, Norway).

### Generation of HLA–Peptide Multimeric Complexes

HLA–B19 peptide multimeric complexes were constructed as previously described [10] and are listed in Table 2. Briefly, recombinant β2 microglobulin and HLA heavy chain (modified by deletion of the transmembrane–cytosolic tail and addition of a BirA enzymatic biotinylation site) were expressed in BL21pLysS *Escherichia coli*. The resulting inclusion bodies were purified and solubilized in urea, and refolded by limiting dilution. Peptides were purchased (Biopeptide, San Diego, California, United States) or synthesized by F-moc chemistry and were of greater than 95% purity. The refolded complex was then concentrated, buffer exchanged, biotinylated with BirA enzyme, and purified by fast protein liquid chromatography. HLA class I peptide complexes were stored at −80 °C. Before staining, the monomers were tetramerized with phycoerythrin or Alexa 647–labeled streptavidin (Molecular Probes, Eugene, Oregon, United States) at a molar ratio of 1:4. In addition, a Pro5 Pentamer containing the HLA-B*40 TEADVQQWL peptide conjugated to allophycocyanin was purchased (ProImmune, Oxford, United Kingdom). Specificity of the MHC monomers was confirmed using T cell lines established in vitro. When tested against non-HLA-matched cytotoxic T lymphocyte (CTL) lines, no population was detected (<0.02%; data not shown).

**Table 2:**
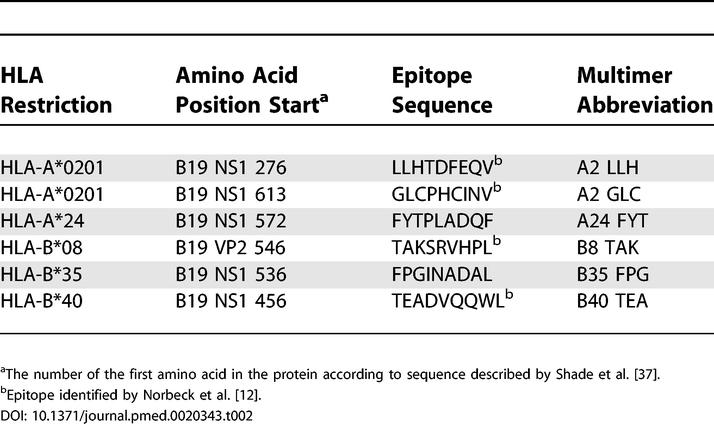
HLA Restriction and Peptide Sequence of B19 Epitope Multimers Used

### FACS Analysis

Cryopreserved PBMCs were thawed and washed twice with RPMI-1640 supplemented with 10% fetal calf serum, L-glutamine, penicillin, streptomycin, and Hepes buffer at pH 7.5. In preliminary experiments, freshly isolated PBMCs were stained in parallel with the same results. PBMCs (2.5 × 10^5^ cells) were stained with the respective major histocompatibility complex multimer and incubated for 20–30 min at 37 °C. After two washes with PBE (2 mM EDTA and 0.05% BSA, in PBS [pH 7.4]), cells were co-stained with the appropriate monoclonal antibodies for 15 min on ice, and fixed in 1%–2% formaldehyde. Monoclonal antibodies used were directly conjugated and purchased ( Becton-Dickinson, Stockholm, Sweden). Four-colour FACS was performed using fluorochrome-coupled anti-human CD3-, CD8-, CD27-, CD28-, CD38-, CD45RA-, CD45RO-, CD57-, CD62L-, CCR7-, and perforin-specific antibodies. For perforin staining, the cells were permeabilized for 15 min using permeabilizing solution (Becton Dickinson, Palo Alto, California, United States) before staining with perforin monoclonal antibody. Cell acquisition was performed with a four-colour FACS by using a FACSCalibur with CellQuest software (Becton Dickinson).

### T Cell Lines and Functional Assays

PBMCs were pulsed with 50 μM of the respective epitope and cultured at 2 × 10^6^ cells/ml in 24-well plates for 12–18 d. At day three, 10 units/ml of IL-2 was added. Half of the medium was replaced each third day with fresh medium containing 10 units/ml of IL-2.

### Ex Vivo IFNγ ELISpots

IFNγ ELISpots were performed as described previously [11]. Briefly 2.5 × 10^5^ PBMCs were stimulated in triplicates with peptide pools/PHA (Sigma, St. Louis, Missouri, United States). Synthetic peptides, 15-mer, overlapping by ten amino acids spanning the entire B19 protein sequence were used in pools of ten at a final concentration of 10 μM. IFNγ responses were confirmed using individual peptides, with optimal epitopes and HLA restriction identified by synthesis of truncated peptides, prepulsing, and extensive washing of HLA-matched and -mismatched target cells, and synthesis of HLA–peptide multimeric complexes [12]. Using these techniques we confirmed previous epitopes in two independent cohorts, and identified two new epitopes shown in Table 2, the nonamer FYT restricted by HLA-A*24 and FPG restricted by HLA-B*35. The latter is in addition to the HLA-B*35 epitope described previously [5].

### Intracellular Cytokine Staining

Short-term stimulation of PBMCs was carried out using either peptide pools or individual peptides as previously described [13]. Epitope HLA restriction required the prepulsing of matched and mismatched PBMCs (from B19 seronegative individuals) with peptide at 20 μM for 1 h at 37 °C. Cells were washed and added to CTLs at a 10:1 ratio for 1 h before the addition of Brefeldin A.

### Chromium Release Assay

CTLs were set up as described above. The cytolysis was performed by killing of chromium-labelled targets as described previously by Nixon et. al. [14] using LBL721.220 transfected with HLA-A*0201 as target cells.

## Results

### B19-Specific CD8^+^ T cells Expand and Persist at High Frequency Following Resolution of Acute Symptomatic Infection

Eleven adults with acute B19 infection (five in Stockholm cohort and six in Oxford cohort) and five remotely infected seropositive individuals were studied. The clinical details, symptom duration, and HLA types are shown in Table 1. Surprisingly, in both cohorts (studied independently) the frequency of B19-specific (multimer-positive) CD8^+^ T cells in peripheral blood samples continued to increase for many months following symptom resolution. Figure 1A shows representative staining from patient O3. The frequency of A24 FYT tetramer-staining CD8^+^ T cells increases up to 15 mo after symptom presentation. Patient O3 presented with rash and arthritis; however, symptoms resolved within 5 wk and clinical examination was entirely normal at the time of the second and subsequent venesections. Figure 1B and 1C show the levels of multimer-positive CD8^+^ T cells for the Oxford and Stockholm cohorts of acutely infected individuals over time. Only one sample was obtained for patient O6; all other individuals were studied 2–7 times. All acutely infected individuals showed B19-specific CD8^+^ T cell percentages ranging from 0.09% to 4.5% total CD8^+^ T cells. The levels rose for at least the first 4 mo and frequently persisted for 12–32 mo after symptom onset. The frequencies at 22 mo (or nearest time point) were significantly greater than at first sampling (Willcoxon rank sum test, *p =* 0.0020), and than those observed for remotely infected seropositive individuals (Figure 1B; Mann–Whitney*U* test, *p =* 0.0022).

**Figure 1:**
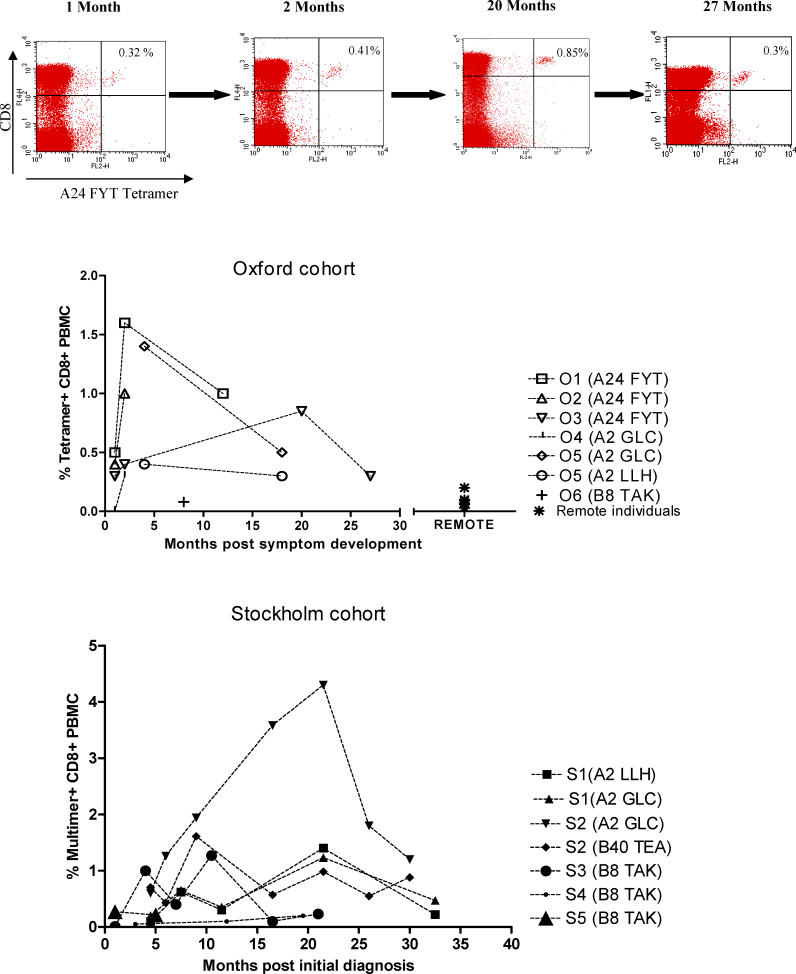
B19-Specific CD8^+^ T Cells Persist at High Levels for Many Months after Acute Infection (A) Representative A24 FYT tetramer staining of individual O3\′s PBMCs. Plots are gated on live CD8^+^ lymphocytes stained directly ex vivo. Percentages shown are those of tetramer-positive CD8^+^ T cells. Time points indicated refer to the number of months after first symptoms reported. Symptoms in this individual lasted 5 wk. (B) Frequency of B19-specific responses over time for six acutely infected individuals in the Oxford cohort (O1–O5) and five remotely infected individuals (OR1–OR5). In one case, two epitopes were studied. (C) Frequency of B19-specific responses over time for five acutely infected individuals in the Stockholm cohort (S1–S5). In two cases, two epitopes were studied.

B19 DNA could be detected by nested PCR in serum for different time periods (see Table 1). No B19 DNA could be amplified from individuals in the remotely infected group. IgG levels rose quickly and were maintained at a stable level in patients studied (data not shown).

### B19-Specific CD8^+^ T Cells Remain Activated Following Resolution of Acute Symptomatic Infection

In order to understand the origins and potential pathogenic role of virus-specific CD8^+^ T cells during and after acute infection, we studied the activation phenotype of these cells ex vivo. Figure 2 shows examples of T cell phenotypic marker expression at early and late sample time points for three different patients using three different B19 peptide–HLA multimers. Figure 2A shows that HLA-B*40 TEA pentamer-positive CD8^+^ T cells from individual S2 are largely perforin positive and CD62L negative, and that these changes become more pronounced from the early (4 mo) to late (21 mo) time points. Figure 3 shows sequential phenotypic data for all of the acutely and remotely infected Oxford cohort. The upper left panel shows that all acutely infected patients showed increases in perforin expression on their B19-specific T cells over the first year following symptom development. By contrast, the seropositive remotely infected individuals showed low levels of perforin expression on tetramer-positive cells. These differences were statistically significant (Mann–Whitney *U* test, *p =* 0.0079). Figure 4 shows sequential data from two individuals, S1 and S2, of the Stockholm cohort, each studied with two different HLA–peptide multimers at five or six time points after acute presentation.

**Figure 2:**
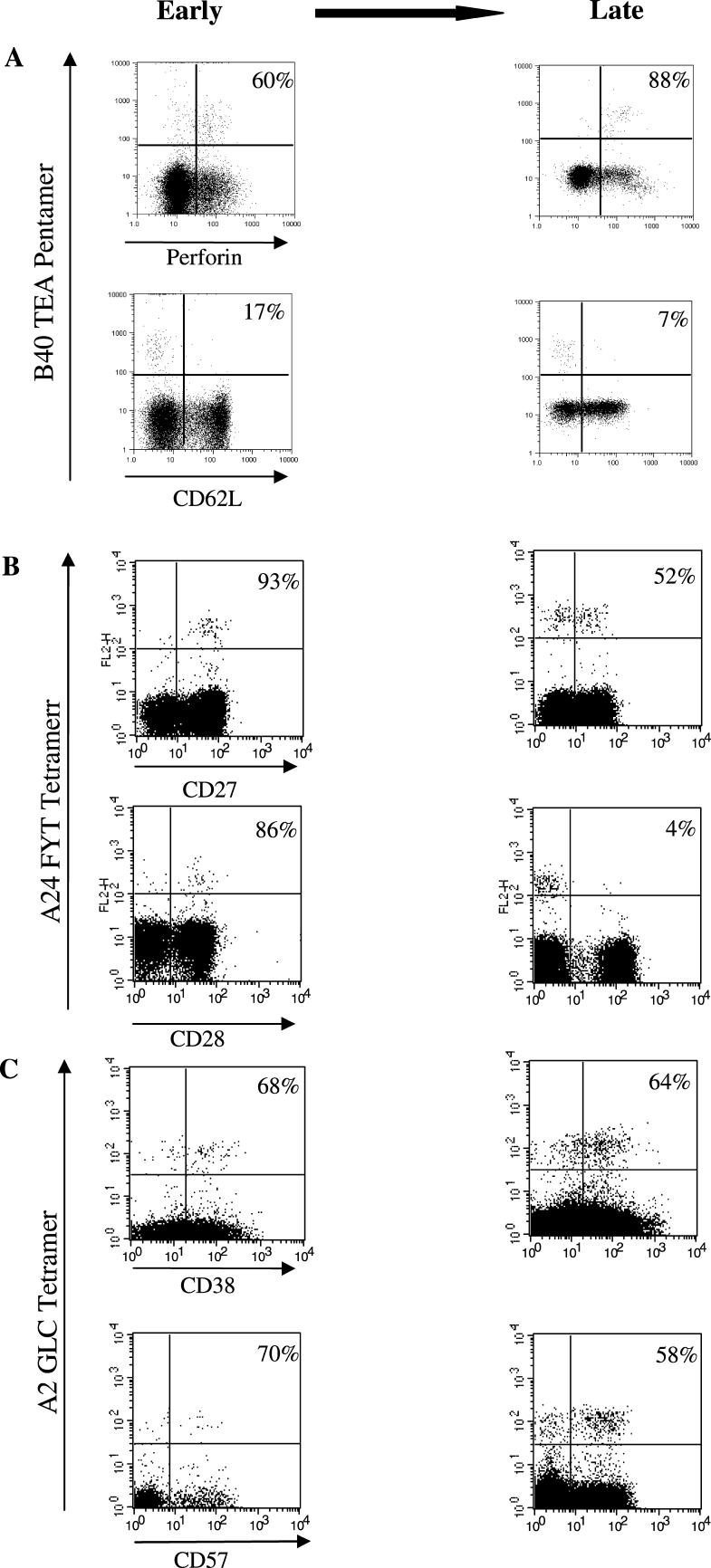
Representative Ex Vivo Phenotyping of B19-Specific CD8^+^ T Cell Populations over Time Percentages shown are the frequency of marker-positive cells amongst multimer-positive cells (plots gated on live CD8^+^ lymphocytes ex vivo). (A) Patient S2 B40 TEA pentamer staining at 4 mo (left) and 21 mo (right), showing perforin and CD62L levels. (B) Patient O3 A24 FYT tetramer staining at 2 mo (left) and 20 mo (right), showing CD27 and CD28 staining. (C) Patient O5 A2 GLC tetramer staining at 4 mo (left) and 18 mo (right), showing CD38 and CD57 staining.

**Figure 3:**
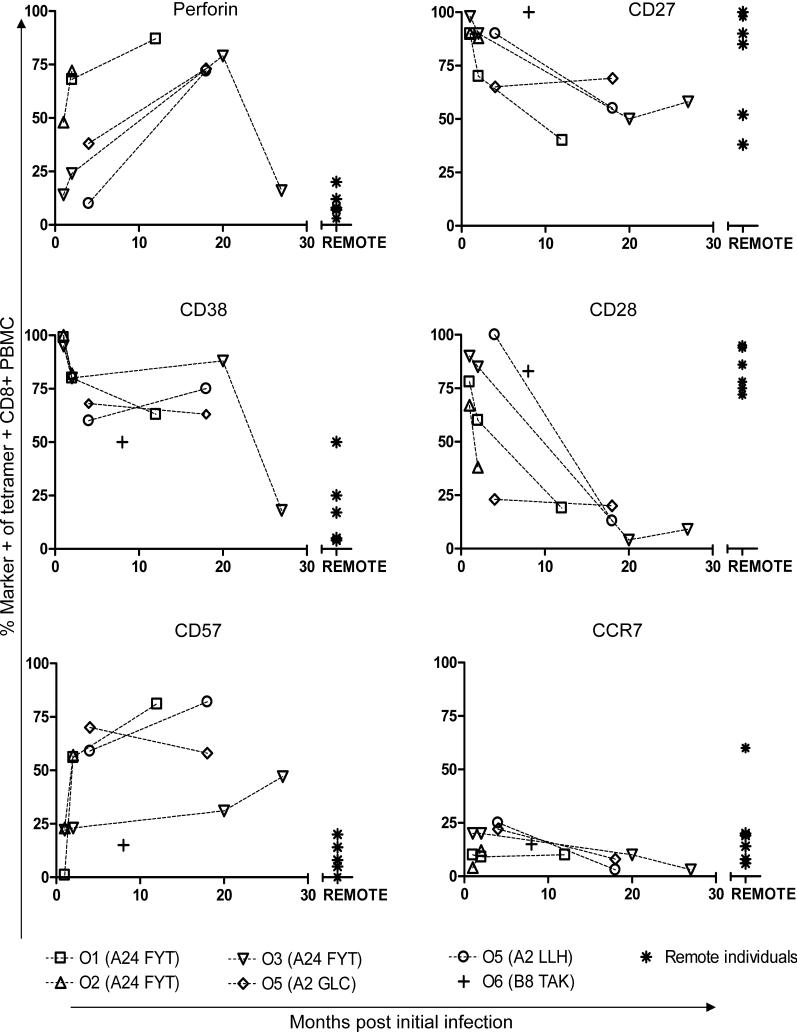
Acutely infected individuals Maintain Activated Mature Effector Cells after Resolution of Acute Infection Remotely infected individuals show a less mature/activated phenotype but express only low levels of CCR7 and CD62L (Oxford cohort). The y-axis show the frequency of marker-positive cells amongst tetramer-positive CD8^+^ cells, while the x-axis show the number of months after symptom onset. Data are shown in perforin, CD38, CD57, CD27, CD28, and CCR7: data are derived from six acutely infected individuals in the Oxford cohort (O1–O3, O5, and O6; insufficient cells available for O4) and five remotely infected individuals (OR1–OR5).

**Figure 4:**
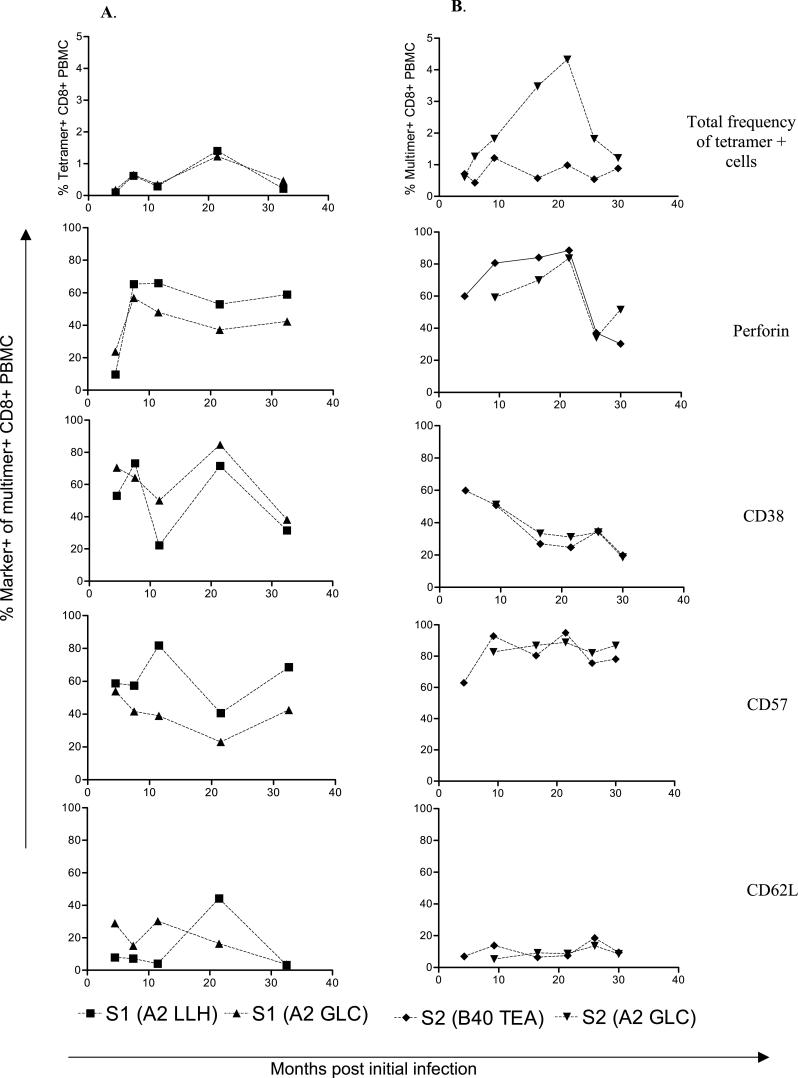
Longitudinal Phenotype Analysis of B19-Specific CD8^+^ T Cell Responses in Acutely Infected Individuals in Stockholm Cohort (A) Blood from patient S1 was stained with the two tetramers A2 LLH and A2 GLC. (B) Blood from patient S2 was stained with tetramers A2 GLC and the Pro5 B40 TEA pentamer. The top panels show the frequency of tetramer-positive cells over time for the two different responses in each individual (data equivalent to those in Figure 1C). The subsequent panels show the frequency of B19-specific CD8^+^ cells positive for perforin, CD38, CD57, and CD62L in both patients.

CD62L expression was found to be low at all time points in the acutely infected cohort as well as in the remotely infected cohort (see Figure 2A, lower panels; Figure 4, bottom panels; data not shown). Figure 3 also shows that, for the Oxford acutely infected cohort, CCR7 expression was low and fell over the study period. B19-specific CD8^+^ T cells from four of the five remotely infected individuals had low CCR7 expression (see Figure 3, bottom right panel).

Figure 2B (upper panels) shows the frequency of CD27 expression on A24 FYT tetramer-positive T cells for patient O3. Figure 3 (upper right panel) shows the data from all of the Oxford cohort. CD27 was downregulated over time in acutely infected individuals. Three of the remotely infected individuals had high levels of CD27 expression on their tetramer-positive populations, while two had slightly lower levels, with only approximately 50% of tetramer-positive cells expressing CD27. B19-specific T cells from the acutely infected individuals also showed downregulation of the CD28 marker over time. This is shown for individual O3 in Figure 2B (bottom panels). Two months after symptom onset, 86% of multimer-positive cells expressed CD28, while at 20 mo only 4% of multimer-positive cells expressd CD28. Figure 3 (middle right panel) shows a similar pattern of CD28 downregulation over time for multimer-positive cells from the Oxford acutely infected cohort. Similar results were seen for the Stockholm cohort (data not shown). By contrast, B19-specific CD8^+^ T cells from remotely infected individuals were predominately CD28^+^. The T cells 20 mo post-infection in acutely infected individuals expressed significantly lower CD28 than in the remotely infected cohort (Mann–Whitney *U* test, *p =* 0.015).

### Persistent CD38 Expression Following B19 Infection Suggests Ongoing Low-Level Antigenic Stimulation

All acutely infected individuals maintained high levels of CD38 expression for more than 10 mo after symptom onset, as shown for the Oxford cohort in Figures 2C and 3 and for two individuals of the Stockholm cohort in Figure 3. Strikingly, for one patient, O3, there was a dramatic fall in CD38 expression at 27 mo compared to the 20-mo time point. Figure 4 shows the sequential CD38 expression of multimer-positive T cells of two specificities for Stockholm patients S1 and S2. S1 shows fluctuations over a period of over 32 mo; S2 shows a gradual decline in CD38 expression from high levels over a comparable period.

CD57 expression levels increased over time in almost all acutely infected individuals. This is shown for one Oxford individual in Figure 2C, for the Oxford acutely infected cohort in Figure 3 (bottom left panel), and for two of the Stockholm cohort in Figure 4. Thus, for individual S2 (Figure 4) a high level of CD57 expression was maintained at 30 mo, the last time point tested. By contrast, Figure 3 (bottom left panel) shows that remotely infected individuals have low levels of CD57 expression on their B19-specific CD8^+^ T cells. T cells 20 mo post-infection in acutely infected individuals expressed significantly higher CD57 than in the remotely infected cohort (Mann–Whitney *U* test, *p =* 0.0043).

### Differing Patterns of CD45 Isoform Expression Follow Acute B19 Infection

CD45 isoform expression showed variation between individuals. Most acutely infected patients from the Stockholm and Oxford cohorts expressed high levels of CD45RO within a few months of symptom onset, with variable downregulation over time (data not shown). In the remotely infected cohort, three individuals expressed high levels of CD45RO on tetramer-positive cells. Two of these individuals, however, had only just over 50% of their tetramer-positive cells expressing CD45O (data not shown). CD45RA was downregulated in some patients in the Oxford cohort, whereas in the Stockholm cohort the B19-specific CD8^+^ cells during the entire study period generally expressed high frequency of CD45RA (data not shown).

### B19-Specific CD8^+^ T Cells Have Efficient Effector Function

Figure 5A shows that PBMCs from patient O1 at the 2-mo time point are capable of immediate IFNγ release on stimulation with the FYTPLADQF peptide. The number of IFNγ-releasing T cells corresponds to approximately 0.3% of CD8 T cells (since at this time point, 40% of PBMCs were CD8^+^). At this time point, 1.6% of CD8^+^ cells were stained with the A24 FYT tetramer (Figure 5A, right panel). Thus, the numbers of cells capable of immediate IFNγ secretion were approximately 5-fold lower than the numbers of tetramer-staining cells. Figure 5B shows that B40-restricted TEA-specific T cells from patient S2 (at 30 mo) were able to proliferate rapidly in vitro on stimulation with peptide. After 12 d, 90% of surviving CD8^+^ T cells were B40 TEA-specific. B19-specific T cell lines were capable of rapid IFNγ production, as assessed by intracellular cytokine release assay (data not shown).

**Figure 5:**
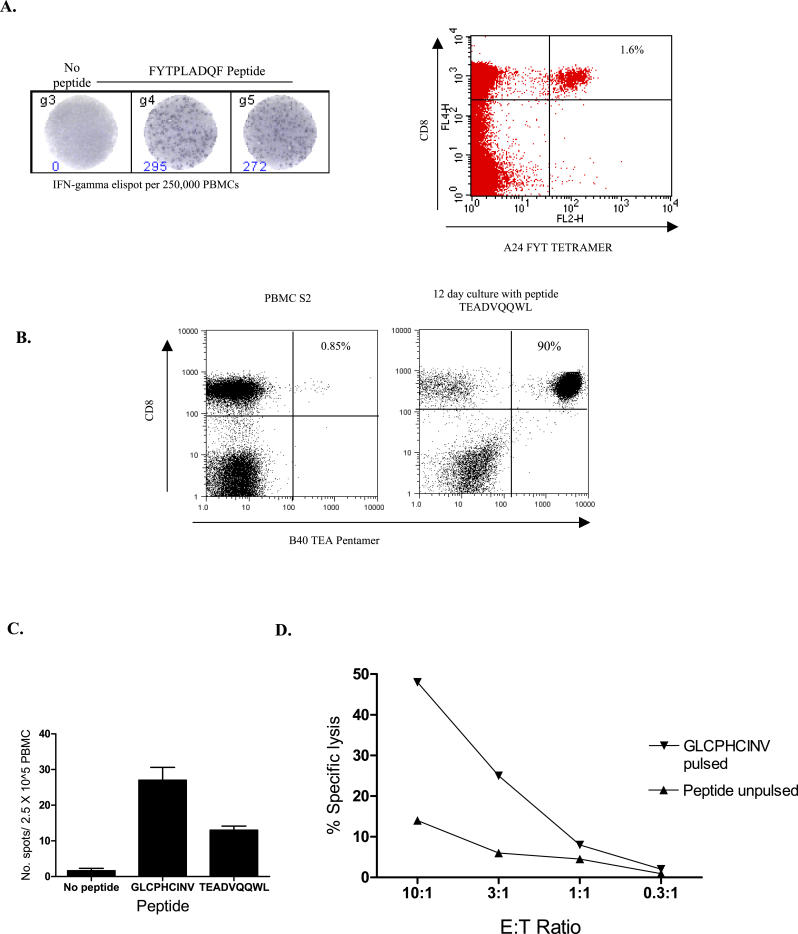
B19-Specific CD8^+^ T Cells Secrete IFNγ Ex Vivo, Proliferate, and Show Cytolytic Function In Vitro (A) Left panel shows that PBMCs from acutely infected patient O1 secrete IFNγ ex vivo after 18 h of FYTPLADQF peptide stimulation. Negative control (zero spots) and two peptide-stimulated wells from an ELISpot plate are shown. Numbers represent IFNγ-secreting cells per 250,000 PBMCs. Right panel shows A24 FYT tetramer staining of PBMCs at same time point, displaying the number of tetramer-positive cells expressed as a percentage of CD8^+^ T cells. (B) Tetramer staining of patient S2\′s PBMCs ex vivo (left) and after short-term TEADVQQWL peptide stimulation in vitro (right). (C) Ex vivo IFNγ ELISpot results for remotely infected individual OR3. Mean and standard deviations of triplicates are shown. Cells were stimulated for 18 h with no peptide, GLCPHCINV, or TEADVQQWL. (D) ^51^Cr release assay using HLA-A2-restricted GLCPHCINV-specific CTLs from individual OR1. PBMCs were stimulated for 14 d with GLCPHCINV peptide and cytolysis was tested against HLA-A*0201-transfected LBL.721.220 target cells at various effector-to-target-cell (E:T) ratios.

Lastly, we studied the function of B19-specific CD8^+^ T cells from remotely infected individuals (who in all cases had no symptoms attributable to possible B19 infection for at least 5 y). Figure 5C shows that these cells were capable of direct ex vivo IFNγ release on 18 h culture with GLCPHCINV or TEADVQQWL peptides. A T cell line derived by culture of OR1 PBMCs with peptide in vitro was capable of HLA-A*0201-restricted B19-peptide-specific cytolysis (Figure 5D).

## Discussion

In this study we observed, to our knowledge for the first time, a striking pattern of evolving, long-lived CD8 immune responses against B19 in 11 adults with primary B19 infection. Although the symptoms of this virus are generally short-lived and the virus is not classically regarded as persistent, the immune responses showed a sustained activated state many months after initial infection. This pattern was observed in a range of patients in two different clinical centres and appears to represent a new and distinct style of host–virus relationship. This is the first example to our knowledge of an “acute” human viral infection inducing persistent activated CD8 T cell responses.

The CD8^+^ T cell responses tracked here were mapped using a comprehensive screening system facilitated by the compact and stable viral genome of B19. The virus has only one NS gene, which appears to be the major target of CD8^+^ T cell responses during acute disease, with a range of epitopes identified [5,12]. HLA-A2-restricted epitopes were commonly targeted, but, interestingly, no clear-cut dominance of one over the other was consistently seen, in contrast to infections such as CMV and HIV [15,16]. CTL responses to HLA-A2 and non-HLA-A2 epitopes showed similar kinetics, frequencies, and phenotypes. Thus, future studies might reasonably track defined epitopes, rather than requiring individual mapping, as is the case in, for example, HCV [17–19].

We used, to our knowledge for the first time, HLA–peptide multimeric complexes to detect CD8^+^ T cell responses during acute B19 infection. Surprisingly, these continued to increase in magnitude at later time points, long after resolution of acute symptoms. In some cases, these responses reached high levels in blood, and were sustained over many months. Even responses of lower frequency appeared to show this delayed expansion. This differs to responses to almost all other human viruses studied in such detail. Responses to HIV and HCV are strong in acute infection, but typically decline as virus is controlled [20]. EBV-specific responses to latent antigens may increase over time [21]. Few data exist on acute CMV infection in immunocompetent humans, but in murine infection, a phenomenon of “memory inflation” is seen for some but not all epitopes [22–24]. Here, responses showed gradual accumulation over time after a short acute response and a lag period of about 8–10 wk after acute infection. The current study did not have the resolution to determine whether B19-specific responses were biphasic in this manner, although in the mouse such phenomena may differ substantially between different virus preparations, doses, and experimental settings.

The B19-specific T cell populations underwent contraction 1.5–2 y after acute infection. The kinetics of this contraction were not defined in this study, but “remotely infected” individuals, who had no recent history of infection, and in some cases may have been primarily infected as long ago as 30 y previously, showed smaller populations of B19-specific T cell populations . However, as we have noted previously, B19-specific CTLs are readily detectable—often much larger than equivalent responses to viruses such as influenza and comparable to some EBV-specific responses [5]. A single individual who had a documented B19 infection 10 y previously showed populations of a size and phenotype (see below) similar to the other remotely infected persons.

In addition to a sustained and prolonged expansion of antiviral responses, we also observed continued maturation of B19-specific CD8^+^ T cells in the cohorts of acutely infected individuals studied. This expansion was consistent across a range of markers, all of which have been linked to the evolution of antiviral “effector” memory cells against persistent virus infections. Consistent with these data is the finding of sustained CD8 effector function of the Swedish cohort over time, as evidenced by IFNγ production in response to viral peptides [12]. Antiviral T cell responses to CMV have been extensively studied and are typically regarded as exhibiting a “mature” phenotype associated with loss of expression of co-stimulatory molecules CD27 and CD28, sustained loss of lymph node homing markers CD62L and CCR7, and generally positive expression of intracellular perforin [7,25–27]. A particular feature of these cells is sustained expression of CD57, which is considered to be a marker of terminally differentiated cells [20]. Although there are substantial differences between individuals, many groups report re-expression of CD45RA on such highly differentiated cells [28–31]. All of these features were clearly reproduced in the B19-specific responses tracked in the months following acute infection. Interestingly, the gradual evolution of responses from a CD28^+^ to CD28^−^ status, in concert with changes in other markers, could be clearly tracked over time. These phenotypic changes have not been extensively investigated in human CMV, but in murine CMV, CD28 loss does appear to occur relatively early, and to be subsequently maintained in the immunodominant populations [32].

It is generally considered likely, although not proven, that such marker evolution represents a maturation pathway, driven by restimulation in vivo with antigen. The nature or duration of the encounter, coupled with the survival of antigen-specific cells, may lead to the typical appearances of T cells specific for different persistent virus infections [7,25]. The findings that not only are the cells phenotypically mature but also strongly activated in vivo (CD38^+^), is consistent with continuous encounter with antigen over the post-infection period. This period of restimulation appears to be sustained, but, unlike in CMV, appears to wane over time, perhaps after 1.5–2 y. Eventual disappearance of antigen would be consistent with the relatively less differentiated phenotype seen in remotely infected individuals. Nevertheless, although such populations are smaller in size, less activated, and less mature, they retain a CD62L-low phenotype. Murine T cell populations that exist in the absence of antigen typically show slow reversion to a CD62L-high state [33], even in the case of CMV [32]. Thus antigenic drive may be reduced but would appear to be still sufficient to maintain an “effector” memory T cell population.

The striking features of the T cell responses to B19 infection indicate persistence of antigen long after the resolution of acute infection. The status of the virus in the post-acute period is not fully understood. Direct nucleic acid analysis has revealed loss of detection in blood after 3 wk in infected volunteers [3], although this may be prolonged in some cases [4]. Nested PCR analysis in our study revealed the presence of B19 DNA in blood at early time points during acute infection, but such assays were negative at time points 6–12 mo after infection, when T cell populations remained activated. It is possible that B19 persists, in the blood and other sites including bone marrow, joints, or skin. Detection of B19 DNA in the bone marrow of asymptomatic remotely infected volunteers has been reported [3]. The finding of parvovirus in skin remains restricted to a single study of a specific genotype distinct from the genotype 1 strains found in our study participants [34]. Low-level, contained replication at a tissue site for weeks or months after infection does seem like the most likely explanation for the immune responses seen, and more sensitive quantitative PCR assays are being validated to address this question. It is also possible that viral antigen is retained, for example, on follicular dendritic cells, following the extremely high burden seen acutely. Alternatively subgenomic particles may be generated in the post-acute period, in the absence of full viral replication. Parvovirus is not thought to establish true latency or integrate into the host genome, so the mechanism behind this low-level persistence remains to be explored.

The relationship between joint or bone marrow pathology and the T cell responses seen is not clear. Indeed, since the most active CD8^+^ T cell responses were seen at stages where joint symptoms had resolved, these T cell responses are unlikely to be directly involved. It remains an open and interesting question, however, whether these or perhaps CD4^+^ T cells are involved in the prolonged inflammatory arthritis seen in a proportion of cases. A previous study did identify an HLA association with B19-induced arthritis syndrome, suggesting a significant role for cellular immune responses [1]. All our patients presented—as is common in adults—with joint symptoms, and in principle an acutely infected but asymptomatic group would provide an ideal comparison to address such a question. It is also possible that the CTL responses seen might be involved in immune-mediated pathology in the bone marrow. Although the virus itself may lead to direct death of the critical progenitor cells, in other settings vigorous T cell responses can also contribute importantly to marrow suppression through lytic and non-lytic pathways [35].

In summary, this is the first demonstration to our knowledge of a virus not thought to cause true or classical persistent infection leading to a persistent activated CD8^+^ T cell response. Responses of this quantity and quality (i.e., CD27 and CD28 low and CD57 and perforin high) have only previously been seen for infection with CMV, a virus known to establish persistent infection. Our data suggest that B19 persists in some form after acute infection, and provokes sustained activated CD8^+^ T cell responses, which might ultimately play a role in viral clearance. Defining the role of CTLs in this setting will be of value not only in expanding further our understanding of the role of T cells in acute and persistent viral infections but also in vaccine design and in immunotherapy, as has been applied to treatment of EBV and CMV infections in immunosuppressed individuals. B19 is a small virus and attracts relatively little attention from clinicians and immunologists [36], but clearly attracts a great deal of attention from the immune system. Now that progress has been made in the definition of the kinetics and antigenic targets of these immune responses, further studies in specific clinical settings where the virus remains a significant problem could readily redress this balance.

**Patient Summary**

**Background**

Parvovirus B19 is a small virus that is very common. Usually it causes mild symptoms of fever and a typical “slapped cheek” rash on the face, which may then spread; in around one in 20 children, and one in two adults it can also cause joint pain. Other, more serious complications occur in people whose red cells do not last as long as usual, for example, people with sickle cell disease; these people can get a severe aplastic anemia—the bone marrow stops making blood completely for a time. The virus can also cause fetal death if a woman contracts it while pregnant.

**Why Was This Study Done?**

It is not clear how the body's defenses—immune system—work to clear this virus. There is some evidence that in some people the virus might continue to divide in the body for a long time after the first infection. The authors wanted to study one part of the immune system—T cells—in people who had just recently been infected with the virus and compare the findings with people who had had the infection a long time previously.

**What Did the Researchers Do and Find?**

They compared findings from two groups of people: 11 who had recently had the infection (six from Oxford and five from Stockholm) and five who had had the virus many years previously. They found that over the year following the infection, one particular type of T cell continued to increase in numbers and responsiveness to the B19 virus, despite the fact that the patients' clinical symptoms had gotten better.

**What Do These Findings Mean?**

It seems the virus remained in the patients' bodies for a considerable time after they appeared to have recovered, and the virus continued to stimulate T cells to respond to it. These results may be useful in designing a strategy to develop a vaccine for this virus.

**Where Can I Get More Information Online?**

The Health Protection Agency in the United Kingdom has a Web page of information on parvovirus:

http://www.hpa.org.uk/infections/topics_az/parvovirus/gen_info.htm

MedlinePlus also has links to further information:

http://www.nlm.nih.gov/medlineplus/ency/article/000977.htm

## Abbreviations

B19: human parvovirus B19
CMV: cytomegalovirus
CTL: cytotoxic T lymphocyte
EBV: Epstein–Barr virus
HCV: hepatitis C virus
HLA: human leukocyte antigen
IFNγ: interferon-γ
PBMC: peripheral blood mononuclear cell
